# Relational coordination in interprofessional teams and its effect on patient-reported benefit and continuity of care: a prospective cohort study from rehabilitation centres in Western Norway

**DOI:** 10.1186/s12913-018-3536-5

**Published:** 2018-09-17

**Authors:** Merethe Hustoft, Eva Biringer, Sturla Gjesdal, Jörg Aβmus, Øystein Hetlevik

**Affiliations:** 1 Centre for Habilitation and Rehabilitation in Western Norway, Haukeland University Hospital, Bergen, Norway; 2 Department of Global Health and Primary Health Care, University of Bergen, Bergen, Norway; 3 Section of Research and Innovation, Helse Fonna Local Health Authority, Haugesund/Stord, Norway; 4 Centre for Clinical Research, Haukeland University Hospital, Bergen, Norway

**Keywords:** Rehabilitation, Continuity of care, Interprofessional relations, Patient-reported outcome measures, Health care survey, Health services research, Relational coordination

## Abstract

**Background:**

Rehabilitation services depend on competent professionals who collaborate effectively. Well-functioning interprofessional teams are expected to positively impact continuity of care. Key factors in continuity of care are communication and collaboration among health care professionals in a team and their patients. This study assessed the associations between team functioning and patient-reported benefits and continuity of care in somatic rehabilitation centres.

**Methods:**

This prospective cohort study uses survey data from 984 patients and from health care professionals in 15 teams in seven somatic rehabilitation centres in Western Norway. Linear mixed effect models were used to investigate associations between the interprofessional team communication and relationship scores (measured by the Relational Coordination [RC] Survey and patient-reported benefit and personal-, team- and cross-boundary continuity of care. Patient-reported continuity of care was measured using the Norwegian version of the Nijmegen Continuity Questionnaire.

**Results:**

The mean communication score for healthcare teams was 3.9 (standard deviation [SD] = 0.63, 95% confidence interval [CI] = 3.78, 4.00), and the mean relationship score was 4.1 (SD = 0.56, 95% CI = 3.97, 4.18). Communication scores in rehabilitation teams varied from 3.4–4.3 and relationship scores from 3.6–4.5. Patients treated by teams with higher relationship scores experienced better continuity between health care professionals in the team at the rehabilitation centre (b = 0.36, 95% CI = 0.05, 0.68; *p* = 0.024). There was a positive association between RC communication in the team the patient was treated by and patient-reported activities of daily living benefit score; all other associations between RC scores and rehabilitation benefit scores were not significant.

**Conclusion:**

Team function is associated with better patient-reported continuity of care and higher ADL-benefit scores among patients after rehabilitation. These findings indicate that interprofessional teams’ RC scores may predict rehabilitation outcomes, but further studies are needed before RC scores can be used as a quality indicator in somatic rehabilitation.

**Electronic supplementary material:**

The online version of this article (10.1186/s12913-018-3536-5) contains supplementary material, which is available to authorized users.

## Background

Interprofessional teamwork is recognised as a cornerstone for both the philosophy and practice of somatic rehabilitation [1]. It emphasises how teams comprising different health care professionals use a shared strategy to work together towards common aims [2]. The need for interprofessional teamwork stems from the complex nature of patients’ health problems and care needs, with teamwork success dependent on collaboration of health care professionals in teams [3–6]. Well-functioning interprofessional teams are expected to have an impact on continuity of care [4]. However, more research is needed to clarify the association between team functioning and outcomes at patient- and system-levels.

Assessing interprofessional team function is a new and challenging task. Relational Coordination (RC) theory is a framework for assessing teamwork that focuses on communication and relationships among health care professionals in a team [7]. Communication in teams depend on the teams’ underlying relationships [8, 9]. RC is defined as a ‘mutually reinforcing process of interaction between communication and relationships carried out for the purpose of task integration [10]. The RC theory argues that for a team to be effectively coordinated, there is a need for shared knowledge and understanding in teams, as well as relationships built on shared goals and mutual respect [9].

Improved continuity of care has been shown to improve health outcomes, efficiency of care and patient satisfaction [11–14]. Most existing research has focused on aspects of personal continuity of care from the care providers’ perspective; for example, the importance of having a care provider that sees the patient over a time period [15–17]. Information exchange between care providers and care management is also important to ensure continuity [11]. However, continuity of care is a complicated concept, as multiple health care providers in teams care for patients with their own expectations and needs [18, 19]. Therefore, it is important to consider the perspective of the patient when investigating aspects of continuity of care in rehabilitation service delivery. Ideally, improved RC in teams should lead to better continuity of care and increased rehabilitation benefits for the patients involved. Currently, there is a gap in knowledge regarding how collaboration among care providers in a team affects continuity of care [19–22]. To gather patient perspectives on continuity of care from a population representing a broad range of diagnostic groups, it is recommended that the generic Nijmegen Continuity Questionnaire (NCQ) is used [23, 24]. The NCQ captures personal continuity as well as continuity within teams and across services [22], and has recently been translated to Norwegian health care settings (NCQ-N) [25].

To our knowledge, no previous studies have investigated the associations between RC in interprofessional teams and patient-reported benefit and experienced continuity of care. Therefore, we assessed associations between communication and relationships in a range of interprofessional teams and patient-reported benefit and continuity of care in somatic rehabilitation centres in Western Norway.

### Aims

The aims of this study were threefold: to measure RC scores in interprofessional teams in seven rehabilitation centres in Western Norway; assess patient-reported benefit and continuity of rehabilitation care, and investigate associations between RC scores and patient-reported benefit and continuity of care.

## Methods

### Study design

This prospective cohort study used data from two surveys of patients in all seven somatic rehabilitation centres in Western Norway. Baseline data were collected from January 2015 to June 2015, with follow-up data collection 1 year later. All patients had a 3–4 week stay at one of the rehabilitation centres in the period between these patient surveys. Patient treatment at the rehabilitation centres is organised in teams and all patients were linked to their treating team. Health care professionals in the rehabilitation centres were surveyed from January 2016 to March 2016.

### Interprofessional rehabilitation team survey

In cooperation with the leaders of the rehabilitation centres, all health professionals (*N* = 167) engaged in working with patients in the centres were invited to participate in the survey. These centres deliver services via interprofessional teams; we identified 16 teams, which were the unit of interest in the present study, according to RC theory [9]. Some healthcare professionals were members of more than one team in the centre in which they worked; these professionals were asked to respond to the survey for each team they worked with. Most healthcare professionals were affiliated with only one team (*n* = 121), 25 had roles in two teams, 13 in three teams and eight worked in four teams. This gave a possible 121 team member responses from healthcare professionals affiliated with more than one team. Therefore, a total of 242 team member responses were invited. Team members were recruited via an e-mail that included information about the project and a link to the RC Survey in Corporater Surveyor version 3.3 (Corporater Inc.). Responses were received from 124 team members (52%), representing 94 different healthcare professionals in 15 interprofessional rehabilitation teams (56% of all employees). Of the responses; 30 were from 19 team members affiliated with two teams, five from members of three teams and six with roles in four teams. One team was missing as no team members responded to the survey and only three patients responded to the questionnaire. The submission of a completed survey was considered provision of consent to participate.

### RC survey

The RC Survey is based on RC theory and is used in both hospital and primary health care settings [14, 26–29]. The survey has been translated into Norwegian language and validated for Norwegian health care settings in a previous study [30]. The survey comprises seven items evaluating interprofessional team function divided into two sub-scales: four communication items (frequency, accuracy, timeliness and problem solving) and three relationship items (shared goals, shared knowledge and mutual respect) [9]. Each item represents a question (e.g. ‘Do health care professionals in this group communicate *frequently* with you about rehabilitation patients?’), with responses on a five point Likert scale (1 = never, 2 = rarely, 3 = occasionally, 4 = often, and 5 = always). A higher score indicates better communication or relationships in the interprofessional team. RC survey communication and relationship subscale scores are derived by calculating the mean scores for each subscale [31]. RC focuses on communication and relationships between roles in the team, rather than between unique individuals [9].

### Patient surveys

#### Participants

Patients aged over 18 years who were accepted for admission to a rehabilitation centre in Western Norway between January and July 2015 were invited to participate in this study (*N* = 2863). In total, 984 patients (34% response rate) accepted the invitation and returned a completed and written consent to participate [32, 33]. The recruitment of patients for the baseline study is fully described in Moen et al. [33]. A 1-year follow-up survey was sent to the 984 participating patients and 705 (25% of those invited at baseline) responded. We excluded 46 patients because of missing *The World Health Organisation Disability Assessment Schedule* (WHODAS 2.0) data, and four cases that education level was not registered. Finally, 655 patients were included in the analyses (Table 1). Eighteen of the 279 patients who did not respond had died.

**Table 1:** Patient characteristics (*N* = 655)

Proportion women, %	62
Age, mean (SD)	
Women	59 (14.0)
Men	63 (11.9)
Education, %	
Elementary school	21
High school	48
University/college	31
Origin of referral, %	
Hospital	35
General practitioner	65
Referral diagnosis, %	
Neoplasms	7
Diseases of the nervous system	12
Diseases of the musculoskeletal system	52
Diseases of the circulatory system	8
Other	21
WHODAS 2.0 global score, mean (SD)	
Women	31.0 (15.12)
Men	27.0 (16.16)

*Abbreviations*: *SD* Standard deviation, *WHODAS 2.0*: World Health Organization Disability Assessment Schedule 2.0

WHODAS 2.0: This scale assesses disability with the global score (0–100) assessed as: 0–4: no functional problems; 5–24: mild functional problems; 25–49: moderate functional problems; 50–95: severe functional problems; and 96–100: total functional loss

#### Data sources

The WHODAS 2.0 global score as reported in the baseline survey was used as an adjustment variable. This is a 36-item generic patient-reported instrument that measures health and disability [34]. The scale gives subscores for patient self-perceived disability in six functional domains: cognition, mobility, self-care, getting along, life activities, and participation [33, 34]. The WHODAS 2.0 global score ranges from 0 to 100 where 5–24 reflects mild functional loss, 25–49 moderate functional loss, 50–95 severe functional loss and 96–100 total functional loss.

Information about whether the patient was referred by a general practitioner (GP) or a hospital physician was collected from the referral letter at baseline, along with referral diagnoses based on the International Statistical Classification of Diseases and Related Health Problems, Tenth Revision (ICD-10). Additionally, Statistics Norway provided data concerning patient education level. We also included questions from the follow-up survey regarding rehabilitation benefits extracted from the PasOpp Survey [35], developed for the Norwegian Institute of Public Health. Patients were asked to assess how their stay in a rehabilitation centre benefitted their overall health, physical health, mental health, management of activities of daily living (ADL) and participation in social activities.

In addition, we used the *NCQ-N* which covers three aspects of continuity: personal, team and cross-boundary continuity [19, 25, 36]. These domains are closely related to informational, management and relational continuity of care [11, 37]. The original NCQ has been used for patients receiving care from multiple providers in both hospital and primary health care settings [12, 15, 38, 39], but this study is the first to use the NCQ-N [25]. The NCQ-N comprises of 28 items that are positively formulated statements concerning different aspects of continuity of care (e.g. personal continuity: care provider knows me well, *‘This care provider knows my medical history very well’)*, scored using a five-point Likert (1 = strongly disagree, 2 = disagree, 3 = neutral, 4 = agree, 5 = strongly agree). A ‘don’t know’ option was also provided, and set as ‘missing’. Subscales for personal continuity (‘the most important health care professional in the rehabilitation centre knows me’) comprising five items and ‘the most important health care professional in the rehabilitation centre shows commitment’ (three items) were derived using the mean scores of the included items. Furthermore, subscales covering team continuity (four items) within the rehabilitation team and cross-boundary continuity (four items) between the rehabilitation centre and the patients’ regular GP were also used. NCQ-N subscales with fewer than two missing items were included in the analyses.

#### Outcome variables

Five items from the PasOpp Survey were used as outcome variables: overall rehabilitation benefits, physical health benefits, mental health benefits, ADL benefits and social participation benefits. Responses were on a five point Likert scale (1 = not at all, 2 = to a lesser extent, 3 = to some extent, 4 = to large extent, 5 = to great extent), with an additional “not applicable” option (set as ‘missing’).

Four *NCQ-N* subscales were used as outcome variables with a continuous scale, ranging from 1 to 5 (5 = best):

- Personal continuity: the most important health care professional in the rehabilitation centre knows me
- Personal continuity: the most important health care professional in the rehabilitation centre shows commitment
- Team continuity: collaboration among health care professionals in teams within somatic rehabilitation centres
- Cross-boundary continuity: collaboration among health care professionals in teams within somatic rehabilitation centres and GPs in the municipality.

#### Explanatory variables

The main explanatory variables in this study were the RC communication and relationships scores, which were calculated for each team and used as continuous variables, from 1(lowest) to 5 (highest).

### Statistical analysis

Descriptive methods were used to analyse sample characteristics. Given the possible intra- cluster correlation between responses from patients treated by the same team, linear mixed effect models were used to investigate associations between patient-reported rehabilitation benefit items (overall rehabilitation benefit, physical benefit, mental health benefit, ADL benefit and social participation benefit) and NCQ-N personal, team and cross-boundary continuity of care items (as outcome variables). The teams’ RC communication and relationship scores were used as explanatory variables. Team allocation was set as the random effect in all models.

For each of the nine outcome variables listed above, four models were estimated using the RC communication subscale as main explanatory variable. First, an unadjusted model (Model 0) containing only the explanatory variable, RC communication subscale and the outcome variables, rehabilitation benefit item scores and NCQ-N subscale scores. Model 1 was adjusted for referral diagnosis (ICD-10) code grouped as: neoplasms, diseases in the nervous system, diseases in the musculoskeletal system, diseases in the circulatory system and others. Model 2 was adjusted for WHODAS 2.0 global score and referral diagnosis. Model 3 was adjusted for WHODAS 2.0 global score, referral diagnosis, sex, age group at the 1 year follow-up (categorised as: < 20, 21–30, 31–40, 41–50, 51–60, 61–70, and > 71), origin of referral (referred by hospital physician or GP) and level of education (categorised as: elementary school, high school, university/college). Similar analyses were repeated with RC relationship scores as the main explanatory variable.

Because of the use of an electronic version of the RC Survey for healthcare professionals, the data retrieved contained no missing values. The level of statistical significance was set as 0.05. All statistical analyses were performed with IBM SPSS for Windows version 23 (IBM Corp., Armonk, NY) [40] and STATA 14 (StataCorp., College Station, TX) [41].

## Results

### RC scores for rehabilitation teams

The mean communication score among healthcare team respondents was 3.9 (standard deviation [SD] = 0.63, 95% confidence interval [CI] = 3.78, 4.00) and the mean relationship score was 4.1 (SD = 0.56, 95% CI = 3.97, 4.18). The communication scores for the rehabilitation teams ranged from 3.4–4.3, and the relationship scores ranged from 3.6–4.5. Table 2 shows an overview of the 15 teams. The rehabilitation centres varied in size, with 5–17 members in each team.

**Table 2:** Characteristics of interprofessional rehabilitation teams and mean (standard deviation) of team communication and relationship scores

Team	Number of team members^a^	Number of patients treated by team	RC Communication Mean (SD)	RC Relationship Mean (SD)
1	8	20	4.3 (0.46)	4.4 (0.45)
2	5	85	4.2 (0.45)	4.5 (0.37)
3	7	19	4.2 (0.39)	4.3 (0.40)
4	7	49	4.2 (0.37)	4.2 (0.52)
5	5	30	4.2 (0.48)	4.3 (0.41)
6	12	59	4.1 (0.62)	4.3 (0.39)
7	17	35	4.1 (0.50)	4.0 (0.46)
8	8	40	3.9 (0.41)	4.3 (0.35)
9	12	60	3.7 (0.79)	3.8 (0.74)
10	5	38	3.7 (0.61)	4.0 (0.63)
11	7	47	3.7 (0.60)	3.8 (0.43)
12	7	8	3.6 (1.07)	3.9 (0.93)
13	8	30	3.6 (0.54)	3.6 (0.47)
14	8	42	3.4 (0.59)	3.9 (0.53)
15	8	43	3.4 (0.49)	3.8 (0.55)
Total	124	605	3.9 (0.63)	4.1 (0.56)

*Abbreviations*: *SD* standard deviation, *RC* relational coordination

^a^Number of team member responses who completed the RC survey

### Patient-reported benefit and continuity of care

The mean overall benefit, physical health and ADL scores were 3.8 (SD = 0.97, 95% CI = 3.73, 3.88), 3.5 (SD = 1.00, 95% CI = 3.45, 3.60) and 3.2 (SD = 1.05, 95% CI = 3.15, 3.32), respectively (Table 3). Team continuity, representing collaboration among rehabilitation team members, had a mean score of 3.7 (SD = 0.82, 95% CI = 3.61, 3.76). Personal continuity mean scores, for the ‘knows me’ and ‘shows commitment’ subscales were 3.0 (SD = 0.86, 95% CI = 2.96, 3.11) and 2.9 (SD = 0.96, 95% CI = 2.76, 2.94), respectively. The cross-boundary continuity mean score for collaboration between the rehabilitation centre and patients’ GPs, was 2.9 (SD = 0.97, 95% CI = 2.81, 3.02).

**Table 3:** Reported benefit and continuity of care among patients at the 1-year follow-up (*N* = 655)

Outcome variables	*n*	Mean (SD)	95% CI
Overall rehabilitation benefit	624	3.8 (0.97)	3.73, 3.88
Physical health benefit	622	3.5 (1.00)	3.45, 3.60
Mental health benefit	532	3.3 (1.11)	3.19, 3.38
Activities of daily living benefit	565	3.2 (1.05)	3.15, 3.32
Social participation benefit	563	3.1 (1.11)	3.01, 3.19
NCQ-N personal continuity (“knows me”)	524	3.0 (0.86)	2.96, 3.11
NCQ-N personal continuity (“shows commitment”)	425	2.9 (0.96)	2.76, 2.94
NCQ-N team continuity (within somatic rehabilitation)	461	3.7 (0.82)	3.61, 3.76
NCQ-N cross boundary continuity (between rehabilitation centres and GP in municipality)	322	2.9 (0.97)	2.81, 3.02

*Abbreviations*: *SD* standard deviation, *CI* confidence interval, *NCQ-N* Nijmegen Continuity Questionnaire, Norwegian version

### Associations between team RC scores and patient-reported benefit and continuity of care

The results presented in Table 4 are derived from the univariate model because adjustments in the models did not lead to improvement of Model 0. Results from the fully adjusted models are shown in the table in the Additional file 1.

**Table 4:** Unadjusted analysis^a^ of patient-reported benefit and continuity of care score associations with communication and relationship sub-scale scores (*N* = 655)

Rehabilitation benefit					
RC Communication	Overall^b^	Physical^c^	Mental^d^	ADL^e^	Social^f^
b	0.26	0.31	0.30	0.29	0.25
95% CI	−0.09, 0.62	−0.06, 0.67	−0.00, 0.61	0.01, 0.58	−0.06, 0.55
*p*-value	0.145	0.097	0.053	0.044	0.112
RC Relationship					
b	0.35	0.35	0.28	0.04	0.06
95% CI	−0.04, 0.73	−0.05, 0.75	− 0.06, 0.61	− 0.28, 0.37	− 0.30, 0.42
*p*-value	0.079	0.083	0.109	0.786	0.751
Continuity of care					
RC Communication	Personal1^g^	Personal2^h^	Team^i^	Cross- boundary^j^	
b	−0.33	−0.40	0.25	−0.35	
95% CI	−0.58, − 0.09	− 0.71, − 0.09	−0.06, 0.56	−0.72, 0.01	
*p*-value	0.008	0.011	0.114	0.056	
RC Relationship					
b	−0.40	−0.50	0.36	− 0.42	
95% CI	−0.67, − 0.13	− 0.83, − 0.16	0.05, 0.68	− 0.80, − 0.04	
*p*-value	0.004	0.004	0.024	0.030	

*Abbreviations: RC* relational coordination, *NCQ-N* Nijmegen Continuity Questionnaire- Norwegian version, *b* unstandardised estimated regression coefficient, *CI* confidence interval, *ADL* activities of daily living

^a^Based on 18 unadjusted linear mixed effects models with either RC communication score or RC relationship scores as the explanatory variable with team allocation set as the random effect in all models

^b^Overall rehabilitation benefit

^c^Physical health benefit

^d^Mental health benefit

^e^Activities of daily living benefit

^f^Social participation benefit

^g^NCQ-N personal continuity (‘knows me’)

^h^NCQ-N personal continuity (‘shows commitment’)

^i^NCQ-N team continuity (within somatic rehabilitation)

^j^NCQ-N cross boundary continuity (between rehabilitation centres and general practitioner in the municipality)

There was a significant association between RC communication in the team the patient was treated by and ADL benefit (b = 0.29, 95% CI = 0.01, 0.58; *p* = 0.044). All other associations between RC scores and patient-reported rehabilitation benefit scores were non-significant, but these showed positive coefficients and most had CIs crossing zero with small margins. Associations of team communication and relationships with patient benefit variables were also tested across sex, age groups, referral diagnosis, and education level (not tabulated); however, no significant group differences were found. There was a positive association between team relationship scores and patient-reported team continuity (b = 0.36, 95% CI 0.05, 0.68; *p* = 0.024), but no significant associations were found regarding communication. Inverse associations were found between communication and relationship scores in teams and both patient-reported personal continuity scales (‘knows me’ and ‘shows commitment’) (Table 4). In addition, there was an inverse association between relationship in teams and cross-boundary continuity (b = − 0.42, 95% CI − 0.80, − 0.04; *p* = 0.030), whereas no associations were found between communication in teams and cross-boundary continuity of care.

## Discussion

This is the first study to investigate prospective associations between communication and relationships in interprofessional teams (measured with the RC Survey), and patient-reported benefit of the rehabilitation stay and experience of continuity of rehabilitation care. Patients treated by teams with higher relationship scores experienced better continuity in the healthcare services they received. However, this study also found that patients reported lower personal continuity of care when treated by teams with higher communication and relationship scores. High relationship scores were associated with lower cross-boundary continuity of care between the rehabilitation centre and the patients’ GPs, as perceived by the patient.

Communication and relationship skills among healthcare professionals are essential for the quality of healthcare delivery [4–6]. Further, strong relationships in teams are expected to contribute to effective service delivery and improved patient health outcomes [42]. Gittell indicated that team functions are strong when the reported RC scores are ≥4 on a five-point scale, which was found for nine of the 15 teams included in this study [10]. An earlier study investigating RC in 23 teams from six somatic hospitals and six psychiatric units in Western Norway found that 14 of 23 teams had a RC score below 3.4, which was the lowest score for rehabilitation teams in the present study [30]. Further, in this previous study, half of the teams showed relationship scores below 3.8, compared with only one rehabilitation team in the present study [30]. The RC scores in this study were also high compared with previous international studies, indicating strong team functions for interprofessional teams in rehabilitation centre in Western Norway [8, 26, 29, 43, 44]. A reason why communication and relationship skills were higher in the present study than in previous studies may be that working in teams is crucial for well-functioning rehabilitation services, and the present study suggests this was implemented as the working environment in these rehabilitation centres.

The patient-reported rehabilitation benefit was moderate in our study, with the highest scores for overall benefit and physical health. Only a significant association between benefit and team functions (as measured by RC score) was found. This contrasted with previous studies that showed positive associations between RC scores and outcomes [8, 14, 26, 27]. However, we observed consistent (but non-significant) associations between patient-reported rehabilitation benefit scores and RC scores. The relatively small variance of RC scores between teams in this study may explain why these associations did not reach statistical significance. Another reason for the lack of significant association between RC scores and benefit outcomes may be that the RC scores did not capture the medical content of the rehabilitation programmes, which may vary independent of team function. Future studies should supplement the RC score with measures of programme content.

An important finding of this study was the association between team relationship skills and patient-reported team continuity. Good relationships among health care professionals develop shared knowledge and skills in teams, and impact continuity of care [3]. Research has also found that strong relationships among team members impacted building rapport with patients treated by the team, and increased patient satisfaction [8, 38, 44]. Our study confirmed that patient experienced increased satisfaction with care when there was shared knowledge, shared goals and mutual respect among team members.

The associations between RC scores and team continuity in this study suggest that patients experienced better relational treatment from the whole team rather than from a single healthcare professional. Several studies have found that personal continuity impacted on patients’ experienced benefit of care [18, 45–49]. However, previous studies also found positive associations between team continuity and improved patient outcomes [16], which is consistent with the finding of this study. Therefore, an inverse association between RC and personal continuity could be considered as a natural consequence of a well-functioning team. However, evaluation of the potential negative effect of reduced personal continuity is a topic for further research.

Seamless transitions between service levels increase patient satisfaction [46–50]. Our finding that better team functioning was associated with lower patient scores for continuity between the rehabilitation centres and primary care was unexpected. The expectation was that strong team functions in rehabilitation services would increase the emphasis on seamless transitions between the centres and the primary care. An explanation for our finding could be that patient respondents tended to over-report negative experiences with cross-boundary continuity, as these were easier to remember (recall bias). Therefore, cross-boundary continuity resulting in seamless transitions might have been overlooked. Another explanation could be that patients who experienced well-functioning teams had higher expectations for cross-boundary continuity, therefore, the inverse association between team RC and patient rating of cross-boundary continuity might be attributable to patients’ disappointment. However, this finding should be interpreted with caution, as the response rate for this subscale was lower than for the other continuity of care subscales (Table 3). Further, more studies are needed to investigate this research question.

### Study strengths and limitations

Strengths of this study included the prospective longitudinal design and the large and comprehensive study population including patients in rehabilitation centres in Western Norway. A main limitation was the low response rate among patients (34%), which might have resulted in selection bias. Although a high response rate was accomplished from baseline to follow-up (73%), only 25% of the total number of patients invited at baseline responded at follow-up, increasing the problem of representability. Unfortunately, there was no information available regarding non-respondents.

The investigation of associations across multiple health care problems and the use of generic survey instruments were further strengths of this study. However, large numbers of ‘don’t know’ for some NCQ-N items meant that these cases were not included in the analysis and might have caused less certain results. Team members responding for more than one team might also have increased the risk for recall bias. In addition, the response rate for the healthcare professionals was relatively low, which might have introduced selection bias. Healthcare professionals with more than one team might also have experienced difficulties in accurately differentiating communication and relationship patterns for their different rehabilitation teams; if so, this would reduce the differences between teams found in RC scores. However, the response rate for team members affiliated with more than one team was relatively low (24%). In general, the RC survey scores did not vary greatly between the teams, reducing the possibility of detecting weaker associations with the outcomes. Further studies are needed to verify these findings.

## Conclusion

Communication and relationships in rehabilitation teams as measured by RC were higher than in comparable studies. This suggests team functioning is a high priority for somatic rehabilitation centres in Western Norway. This study found a positive association between RC relationship in the team the patients were treated by and team continuity reported by patients. However, we did not show that stronger RC team functions in rehabilitation centres predicted better patient outcomes, with the exception of a significant positive association with improved ADL. The negative associations found between team function within rehabilitation centres and cooperation with primary care should be further studied, as further rehabilitation benefits depend on follow-up in primary care.

## Additional file

Additional file 1: Linear mixed effect models, fully adjusted. (DOCX 24 kb)

## Abbreviations

ADL: Activity of daily living
CI: Confidence interval.
GP: General practitioner
NCQ: Nijmegen Continuity Questionnaire
NCQ-N: Norwegian version of the Nijmegen Continuity Questionnaire
RC: Relational coordination
SD: Standard deviation
WHODAS 2.0: World Health Organization Disability Assessment Schedule 2.0
