# Supplementary material for: Relational coordination in interprofessional teams and its effect on patient-reported benefit and continuity of care: a prospective cohort study from rehabilitation centres in Western Norway
Source: BMC Health Serv Res. 2018 Sep 17;18:719. doi: 10.1186/s12913-018-3536-5 (PMC6142375; doi:10.1186/s12913-018-3536-5)
Supplement: Supplementary file 1 — Linear mixed effect models, fully adjusted. (DOCX 24 kb) [file 12913_2018_3536_MOESM1_ESM.docx]

**Additional file 1.** Patient-reported benefit and continuity of care score associations with communication and relationship subscales scores in Western Norway. Team allocation is set as the random effect in all models (N=655).

|  | **Model: 0** | | | | | **Model: 1** | | | | | | **Model: 2** | | | | | | **Model: 3** | | | | |
| --- | --- | --- | --- | --- | --- | --- | --- | --- | --- | --- | --- | --- | --- | --- | --- | --- | --- | --- | --- | --- | --- | --- |
|  | **Parameter estimation** | | | **Model fit** |  | **Parameter estimation** | | | **Model fit**  **(Comparison with Model 0)** | |  | **Parameter estimation** | | | **Model fit**  **(Comparison with Model 1)** | |  | **Parameter estimation** | | | **Model fit**  **(Comparison with Model 2)** | |
| **RC Communication** | **b** | **95% CI** | **p** | **AIC** |  | **b** | **95% CI** | **p** | **AIC** | **p** |  | **b** | **95% CI** | **p** | **AIC** | **p** |  | **b** | **95% CI** | **p** | **AIC** | **p** |
| Overall rehabilitation benefit | 0.26 | −0.09, 0.62 | 0.145 | 1606.9 |  | 0.27 | −0.11, 0.65 | 0.164 | 1610.9 | 0.415 |  | 0.23 | −0.13, 0.60 | 0.210 | 1609.0 | 0.048 |  | 0.27 | −0.10, 0.65 | 0.152 | 1614.1 | 0.167 |
| Physical health benefit | 0.31 | −0.06, 0.67 | 0.097 | 1622.1 |  | 0.31 | −0.06, 0.67 | 0.096 | 1627.9 | 0.696 |  | 0.24 | −0.09, 0.58 | 0.149 | 1620.4 | 0.002 |  | 0.27 | −0.08, 0.62 | 0.132 | 1620.7 | 0.039 |
| Mental health benefit | 0.30 | −0.00, 0.61 | 0.053 | 1506.9 |  | 0.26 | −0.07, 0.58 | 0.118 | 1512.9 | 0.724 |  | 0.22 | −0.11, 0.54 | 0.187 | 1512.1 | 0.096 |  | 0.32 | −0.02, 0.65 | 0.064 | 1516.6 | 0.139 |
| Activities of daily living benefit | 0.29 | 0.01, 0.58 | 0.044 | 1538.7 |  | 0.28 | −0.03, 0.58 | 0.073 | 1545.1 | 0.808 |  | 0.25 | −0.06, 0.55 | 0.117 | 1545.8 | 0.267 |  | 0.27 | −0.04, 0.58 | 0.089 | 1542.7 | 0.012 |
| Social participation benefit | 0.25 | −0.06, 0.55 | 0.112 | 1584.6 |  | 0.20 | −0.12, 0.52 | 0.226 | 1589.0 | 0.793 |  | 0.16 | −0.16, 0.48 | 0.329 | 1589.4 | 0.210 |  | 0.20 | −0.13, 0.54 | 0.238 | 1589.7 | 0.040 |
|  |  |  |  |  |  |  |  |  |  |  |  |  |  |  |  |  |  |  |  |  |  |  |
| NCQ-N personal continuity (‘knows me’) | −0.33 | −0.58, -0.09 | 0.008 | 1235.3 |  | −0.33 | −0.59, −0.07 | 0.014 | 1241.8 | 0.834 |  | −0.31 | −0.57, −0.05 | 0.021 | 1242.5 | 0.246 |  | −0.30 | −0.57, −0.03 | 0.027 | 1247.0 | 0.142 |
| NCQ-N personal continuity (‘shows commitment’) | −0.40 | −0.71, -0.09 | 0.011 | 1092.2 |  | −0.44 | −0.76, −0.12 | 0.008 | 1095.8 | 0.351 |  | −0.42 | −0.74, −0.10 | 0.010 | 1096.7 | 0.301 |  | −0.37 | −0.70, −0.04 | 0.026 | 1100.2 | 0.106 |
| NCQ-N team continuity (between providers within somatic rehabilitation) | 0.25 | −0.06, 0.56 | 0.114 | 1035.8 |  | 0.16 | −0.13, 0.45 | 0.282 | 1036.8 | 0.136 |  | 0.16 | −0.13, 0.45 | 0.290 | 1038.8 | 0.887 |  | 0.22 | −0.09, 0.52 | 0.163 | 1040.2 | 0.054 |
| NCQ-N cross boundary continuity (between providers within somatic rehabilitation and GP in municipality) | −0.35 | −0.72, 0.01 | 0.056 | 833.5 |  | −0.34 | −0.72, 0.05 | 0.090 | 840.1 | 0.848 |  | −0.35 | −0.74, 0.04 | 0.082 | 841.9 | 0.645 |  | −0.39 | −0.80, 0.01 | 0.056 | 851.3 | 0.470 |
|  |  |  |  |  |  |  |  |  |  |  |  |  |  |  |  |  |  |  |  |  |  |  |
| **RC Relationship** | **b** | **95% CI** | **p** | **AIC** |  | **b** | **95% CI** | **p** | **AIC** | **p** |  | **b** | **95% CI** | **p** | **AIC** | **p** |  | **b** | **95% CI** | **p** | **AIC** | **p** |
| Overall rehabilitation benefit | 0.35 | −0.04, 0.73 | 0.079 | 1606.2 |  | 0.35 | −0.05, 0.75 | 0.083 | 1610.2 | 0.402 |  | 0.30 | −0.09, 0.69 | 0.127 | 1608.5 | 0.054 |  | 0.36 | −0.04, 0.75 | 0.079 | 1613.4 | 0.157 |
| Physical health benefit | 0.35 | −0.05, 0.75 | 0.083 | 1622.0 |  | 0.36 | −0.03, 0.74 | 0.067 | 1627.6 | 0.661 |  | 0.27 | −0.08, 0.62 | 0.131 | 1620.4 | 0.002 |  | 0.36 | −0.00, 0.72 | 0.051 | 1619.7 | 0.028 |
| Mental health benefit | 0.28 | −0.06, 0.61 | 0.109 | 1508.1 |  | 0.24 | −0.10, 0.59 | 0.169 | 1513.4 | 0.614 |  | 0.18 | −0.17, 0.53 | 0.308 | 1512.8 | 0.106 |  | 0.33 | −0.04, 0.70 | 0.078 | 1516.9 | 0.125 |
| Activities of daily living benefit | 0.04 | −0.28, 0.37 | 0.786 | 1542.6 |  | 0.03 | −0.29, 0.34 | 0.871 | 1548.2 | 0.670 |  | −0.03 | −0.35, 0.30 | 0.869 | 1548.2 | 0.159 |  | 0.09 | −0.25, 0.42 | 0.618 | 1545.3 | 0.013 |
| Social participation benefit | 0.06 | −0.30, 0.42 | 0.751 | 1585.0 |  | 0.00 | −0.34, 0.34 | 0.998 | 1590.4 | 0.636 |  | −0.06 | −0.40, 0.29 | 0.739 | 1590.2 | 0.139 |  | 0.09 | −0.29, 0.46 | 0.647 | 1590.9 | 0.044 |
|  |  |  |  |  |  |  |  |  |  |  |  |  |  |  |  |  |  |  |  |  |  |  |
| NCQ-N personal continuity (‘knows me’) | −0.40 | −0.67, −0.13 | 0.004 | 1234.0 |  | −0.38 | −0.65, −0.10 | 0.007 | 1240.7 | 0.870 |  | −0.35 | −0.63, −0.07 | 0.016 | 1241.9 | 0.362 |  | −0.33 | −0.62, −0.03 | 0.028 | 1247.0 | 0.169 |
| NCQ-N personal continuity (‘shows commitment’) | −0.50 | −0.83, −0.16 | 0.004 | 1090.5 |  | −0.49 | −0.84, -0.15 | 0.005 | 1094.9 | 0.470 |  | −0.47 | −0.82, −0.12 | 0.008 | 1096.3 | 0.429 |  | −0.44 | −0.80, −0.08 | 0.017 | 1099.4 | 0.095 |
| NCQ-N team continuity (between providers within somatic rehabilitation) | 0.36 | 0.05, 0.68 | 0.024 | 1034.3 |  | 0.31 | 0.04, 0.58 | 0.025 | 1034.2 | 0.090 |  | 0.31 | 0.03, 0.58 | 0.028 | 1036.8 | 0.960 |  | 0.36 | 0.07, 0.65 | 0.016 | 1037.7 | 0.057 |
| NCQ-N cross boundary continuity (between providers within somatic rehabilitation and GP in municipality) | −0.42 | −0.80, −0.04 | 0.030 | 832.5 |  | −0.41 | −0.80, −0.02 | 0.042 | 838.9 | 0.806 |  | −0.44 | −0.84, −0.04 | 0.032 | 840.4 | 0.480 |  | −0.49 | −0.91, −0.07 | 0.023 | 849.8 | 0.477 |

Abbreviations: RC: relational coordination; NCQ-N: Nijmegen Continuity Questionnaire, Norwegian version; WHODAS 2.0: World Health Organization Disability Assessment Schedule 2.0; b: unstandardised estimated regression coefficient; AIC: akaike information criterion; CI: confidence interval

Model 0: Crude estimates

Model 1: Adjusted for referral diagnosis, grouped as: neoplasms, diseases of the nervous system, diseases of the musculoskeletal system, diseases of the circulatory system, other

Model 2: Adjusted for referral diagnosis, grouped as: neoplasms, diseases of the nervous system, diseases of the musculoskeletal system, diseases of the circulatory system, other and WHODAS 2.0 global score (0–100), assessed as: 0–4: no functional problems; 5–24: mild functional problems; 25–49: moderate functional problems; 50–95: severe functional problems; and 96–100: total functional loss..

Model 3: Adjusted for referral diagnosis, grouped as: neoplasms, diseases of the nervous system, diseases of the musculoskeletal system, diseases of the circulatory system, other and WHODAS 2.0 global score (0–100), age group (categorised as: <20, 21–30, 31–40, 41–50, 51–60, 61–70, and >71), sex, level of education (categorised as: elementary school, high school, university/college) and origin of referral (referred by hospital physician or GP).
